# Decoding Sequence Learning from Single-Trial Intracranial EEG in Humans

**DOI:** 10.1371/journal.pone.0028630

**Published:** 2011-12-09

**Authors:** Marzia De Lucia, Irina Constantinescu, Virginie Sterpenich, Gilles Pourtois, Margitta Seeck, Sophie Schwartz

**Affiliations:** 1 Department of Radiology, Vaudois University Hospital Center and University of Lausanne, Lausanne, Switzerland; 2 Department of Neuroscience, University of Geneva, Geneva, Switzerland; 3 Geneva Neuroscience Center, University of Geneva, Geneva, Switzerland; 4 Department of Experimental Clinical and Health Psychology, University of Ghent, Ghent, Belgium; 5 Department of Clinical Neurology, Geneva University Hospitals, Geneva, Switzerland; 6 Swiss Center for Affective Sciences, University of Geneva, Geneva, Switzerland; 7 Electroencephalography Brain Mapping Core, Center for Biomedical Imaging, Lausanne, Switzerland; Katholieke Universiteit Leuven, Belgium

## Abstract

We propose and validate a multivariate classification algorithm for characterizing changes in human intracranial electroencephalographic data (iEEG) after learning motor sequences. The algorithm is based on a Hidden Markov Model (HMM) that captures spatio-temporal properties of the iEEG at the level of single trials. Continuous intracranial iEEG was acquired during two sessions (one before and one after a night of sleep) in two patients with depth electrodes implanted in several brain areas. They performed a visuomotor sequence (serial reaction time task, SRTT) using the fingers of their non-dominant hand. Our results show that the decoding algorithm correctly classified single iEEG trials from the trained sequence as belonging to either the initial training phase (day 1, before sleep) or a later consolidated phase (day 2, after sleep), whereas it failed to do so for trials belonging to a control condition (pseudo-random sequence). Accurate single-trial classification was achieved by taking advantage of the distributed pattern of neural activity. However, across all the contacts the hippocampus contributed most significantly to the classification accuracy for both patients, and one fronto-striatal contact for one patient. Together, these human intracranial findings demonstrate that a multivariate decoding approach can detect learning-related changes at the level of single-trial iEEG. Because it allows an unbiased identification of brain sites contributing to a behavioral effect (or experimental condition) at the level of single subject, this approach could be usefully applied to assess the neural correlates of other complex cognitive functions in patients implanted with multiple electrodes.

## Introduction

In functional neuroimaging studies, the application of machine learning techniques has recently become a popular method for decoding stimulus-related information at the level of the single response to external stimuli [1], [2]. Most of the machine learning techniques applied to neuroimaging data are intrinsically multivariate and therefore particularly suitable when the main differences between experimental conditions are not in the strength of the activity at specific brain regions, but rather in the configuration or relative spatial locations of simultaneously activated areas (for reviews see [3]–[7]). Indeed, recent applications of machine learning techniques in hemodynamic (e.g. [8]–[13]) and electrophysiological (e.g. [14]–[17]) studies have emphasized the role of widespread activation patterns underlying many human cognitive functions, shifting the focus from the description of specialized brain regions to a network perspective. In addition, because these methods typically exploit high-dimensional data, they often offer a valid alternative to the preselection of a subset of the available data prior to the analysis. In particular, electrophysiological studies often involve the *a priori* selection of specific electrodes prior to statistics, thus severely limiting the possibility to reveal robust effects outside regions of interest and preventing the investigation of spatially distributed effects. In intracranial electroencephalography studies in humans, the use of a very small subset of the data, i.e. highly localized recording sites, is preferred because it gets around the problem of variations in the patterns of surgical implantation across patients (e.g. [18]–[20]).

Here we propose a machine learning approach for the analysis of human iEEG which takes advantage of multiple recording sites and high temporal resolution of brain activity. Following a classification scheme, we aim at demonstrating the feasibility of this method in capturing learning-related changes during a visuo-motor task performed over two sessions.

We recorded iEEG from distributed cerebral locations in two epileptic patients to identify spatio-temporal changes in neural activity related to visuo-motor learning. To characterize the neural activity during the execution of a trained sequence (as compared to an untrained sequence), we employed a decoding algorithm that classifies single-trial data by exploiting learning-related changes in distributed patterns of neural activity. We then estimated which electrodes mostly contributed to the discrimination power in order to identify brain regions implicated in learning-related changes. This approach is challenging because it aims at extracting distinctive features of neural activity during a complex cognitive paradigm at the single-trial level and with minimal *a priori* constraints (for similar approaches in scalp EEG see [17], [21]–[23]). This effort is counterbalanced by the benefit of a statistical assessment of datasets from single individuals, and by taking into account unlocked activity that is overlooked when averaging peri-stimulus epochs as in standard univariate approaches. This approach would thus be particularly appropriate for the study of clinical cases with heterogeneous anatomical and functional characteristics.

The present results show that the proposed multivariate algorithm can track learning-related changes, provides consistent results in two distinct patients analyzed independently, and offers the possibility to estimate in an unbiased way which neural locations may critically contribute to motor sequence learning.

## Materials and Methods

### Experimental Paradigm

#### Ethics Statement

Patients provided written informed consent to participate in this study, which was approved by the ethical committee of the Geneva University Hospitals.

#### Patients description

**Figure 1:**
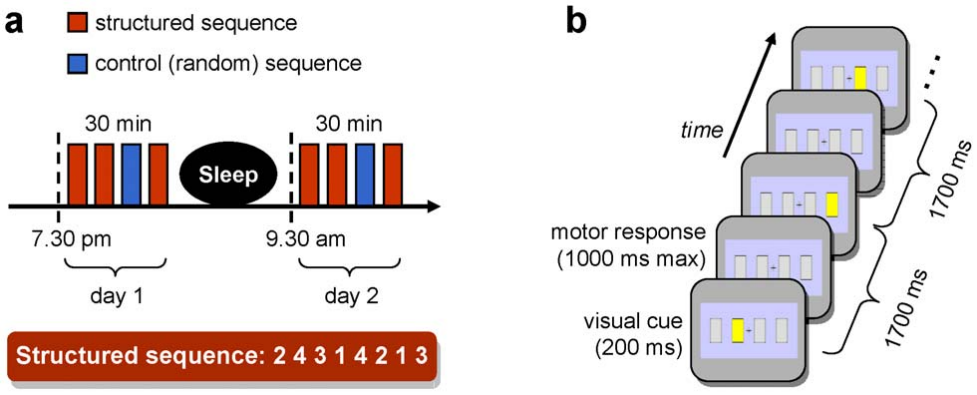
Illustration of the experimental procedure. (a) The experimental protocol included one training session on day one, followed by one night of sleep, and a test session on day 2. Each session lasted about 30 min and comprised 60 S-sequence (3 blocks) and 20 C-sequences (1 block). The task was performed with the non-dominant hand and the trained 8-item sequence is shown on the bottom: finger 1 corresponds to the index and finger 4 to the little finger. (b) On each trial in the sequence, one of the four grey rectangles briefly flashed (yellow during 200 ms), indicating which button to press next. One sequence in this serial reaction time task required 8 keypresses in a specific order.

We tested epileptic patients who had depth-electrodes implanted in several brain regions for presurgical evaluation purposes. Here we report the findings in two patients, patient M.R. (male, left-handed, aged 35) and patient C.S. (female, right-handed, aged 25) who completed the experimental protocol without any major epileptic activity over the whole period of recording (about 18 hours over 2 days, see Figure 1a). During the whole experimental protocol, including one night of sleep, both patients were free of any medication. The ictal semiology consisted of generalized seizures alternating with partial seizures for patient M.R and of complex partial seizures for patient C.S. None of the patients had any detectable hippocampal damage (including sclerosis): the magnetic resonance imaging (MRI) examination was normal for patient M.R and showed bilateral occipital periventricular heterotopia without any hippocampal abnormality for patient C.S. Both patients had a good general clinical status, no cognitive impairment, and no history of sleep disorders. None of the patients had formerly exerted any activity that could influence performance on the main experimental task (SRTT, see below), such as playing a musical instrument or extensive practice with typing on a keyboard. The patients could execute the task without difficulty (see Results). Both patients judged the quality of the night's sleep during the experiment as good (St Mary's Hospital Sleep Questionnaire and verbal interview) and polysomnographic sleep scoring showed good sleep efficiency (see Material S1).

### Behavioral task and experimental procedure

The patients were tested on a serial reaction time task (SRTT) [24], [25]. During testing, the patients faced a computer screen (19 inches) displaying four grey rectangles (2.15×4.30 degrees of visual angle, each) arranged horizontally next to each other (2.15° gaps) on a homogeneous, light blue background, with a fixation cross at the center of screen (Figure 1b). The four rectangles and the fixation cross remained on the screen throughout the experiment. On each trial, one of the four grey rectangles briefly changed its color to yellow for 200 ms. The patients had to react as quickly and accurately as possible by pressing on a response-pad the key that spatially corresponded to the flashed position within a 1000 ms interval. The patients responded with the four fingers of their non-dominant hand (right for M.R. and left for C.S.). Whenever they did not press the correct key or pressed no key within the response interval, an error feedback was displayed (background color turning to dark blue during 200 ms). The next trial started 500 ms after the end of the response period. The inter-stimulus interval was 1700 ms. On each trial in the sequence, the reaction time (RT) and response accuracy were recorded. The SRTT was programmed using the E-Prime software (Psychology Software Tools, Inc., Pittsburg, PA) to allow high temporal precision for stimulus presentation, RT data collection, and marking of the events on the iEEG recording. Two types of sequences were presented: a structured (S) sequence, requiring eight keypresses in a specific pre-determined order and a control (C) pseudo-random sequence. For both patients, the S-sequence used was 2-4-3-1-4-2-1-3, where 1 refers to the left-most rectangle and 4 to the right-most rectangle on the screen. The C-sequence consisted of series of eight keypresses (as for the S-sequence) formed by randomly shuffling two sub-sequences of the 1-2-3-4 positions. Hence, the two finger sequences differed but shared a main global feature: the four distinct positions were flashed once in each of the two successive quartets forming an 8-position sequence. A short pause of 2500 ms was inserted after the last trial of each sequence, i.e. after 8 keypresses. The patients were not informed about the regularity of the S-sequence.

The patients were tested during two sessions on two successive days, including one night of sleep between the sessions (Figure 1a). On day 1, a training session started at 7.45 p.m. and lasted about 30 min. On day 2, a test session started at 9.30 a.m. and lasted 30 min. Each session consisted of three blocks of the S-sequence (20 repetitions each) and one equivalent block of the C-sequence. The C-sequence was introduced in both sessions to allow the assessment of sequence-specific changes in behavior and neural activity, independent of non-specific improvements in executing the visuomotor task. The C-sequence was performed after two S-blocks to ensure that the patients had acquired good practice with the SRT task. The C-block was also followed by a last S-block to limit the interference of the C-sequence on the consolidation of the S-sequence. To obtain information about explicit knowledge of the S-sequence, we asked the patients at the end of the second session on day 2 whether they had noticed any regularity regarding the succession of the positions (or key presses). We also invited them to reproduce the motor sequence on the keypad.

### Intracranial EEG recording

**Figure 2:**
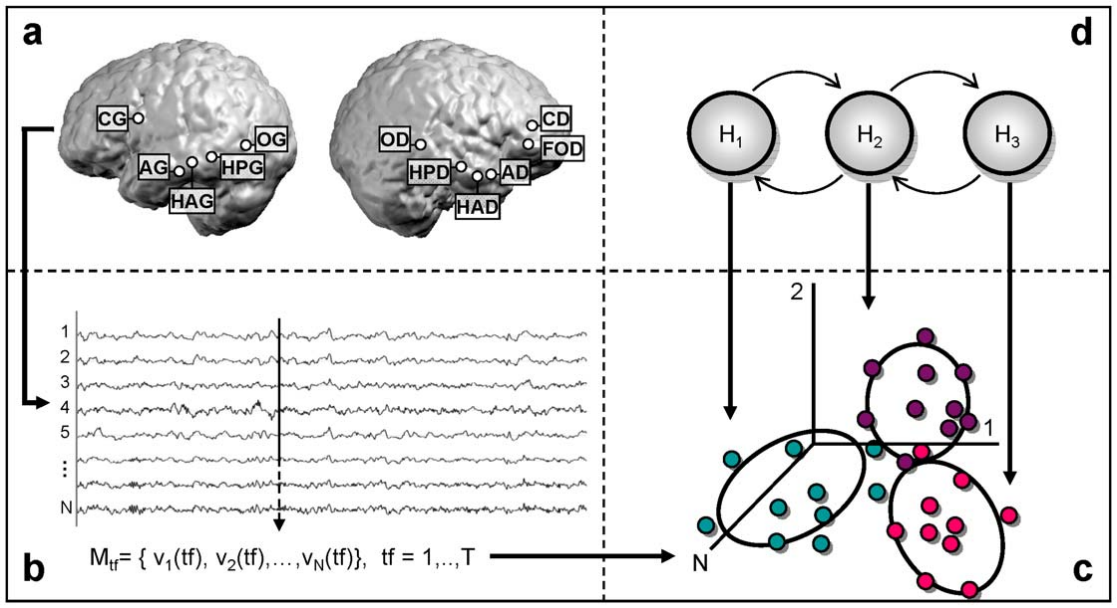
Classification procedure. (a) Representation of the implanted areas in patient C.S, lateral view of both hemispheres; (b) intracranial EEG (iEEG) recorded over the N contacts at each time-frame form an N-dimensional vector; (c) iEEG measures are pooled together within an N-dimensional space irrespective of their temporal order and a GMM is used to estimate Q Gaussian parameters (here Q = 3); (d) a Hidden Markov Model with Q hidden states, initialized by the GMM parameters, is used to update the Gaussian estimations as well as the transition matrix between the hidden states.

We tested both patients while they underwent continuous long-term iEEG recordings. Electrophysiological activity was recorded over arrays of depth electrodes surgically implanted to identify the epilepsy focus. Intracranial EEG was recorded (Ceegraph XL, Biologic System Corps.) using electrode arrays with 8 stainless contacts each (AD-Tech, electrode diameter: 3 mm, inter-contact spacing: 10 mm), orthogonally implanted in several brain regions. Patient M.R. had 64 contacts (8 arrays), covering, bilaterally, the anterior and posterior hippocampal regions, the amygdala, and the fronto-orbital cortex. For patient C.S., a total of 88 contacts (11 arrays) covered anterior and posterior hippocampal regions, the amygdala, the frontal-caudate area, and the occipital cortex, bilaterally, as well as the right fronto-orbital cortex (Figure 2a). For each patient, we determined precise electrode location by the co-registration of a post-operative computed tomography scan (CT) with a high-resolution anatomical MRI. For the iEEG recordings, the reference in both patients was a scalp electrode, subcutaneously implanted, located at position Cz and the ground was another scalp electrode at position FCz of the 10–20 international EEG system. We sampled the iEEG signal at 512 Hz and band-pass filtered between 0.1–200 Hz. We applied DC correction and a 50 Hz notch filter to the data. Intracranial EEG was recorded continuously during the training period on day 1 and testing period on day 2, as well as during the night between the two sessions. Electrooculography using two electrodes placed on the eyes' external canthi and electromyography using chin muscle electrodes were also performed during the sleep periods. Sleep data were scored manually by two trained sleep scorers on 30 s epochs based on Rechtschaffen and Kales standard scoring criteria [26].

### Data analysis

We first analyzed iEEG data from all implanted regions using a classification method based on single-trial responses. This approach aims at classifying single trials of iEEG (epochs corresponding to single key presses) in the S-sequence as belonging to day 1 or to day 2. We then performed a localization procedure to detect which areas contributed significantly to the classification accuracy.

We implemented a multivariate approach to investigate the consolidation of a sequential motor skill based on distributed brain signals. This method presents several advantages for the analysis of intracranial recordings with respect to classic average event-related potentials. First, it exploits single-trial measurement of a different duration therefore accounting for possible latency shifts in the electrophysiological response to similar stimuli and that are not time-locked to the visual cue or motor response. Second, it provides classification estimations for different conditions, whose accuracy can be tested statistically. Finally, by a recurrent procedure it allows to assess the contribution of each measurement site to the classification accuracy. The distinctive features of this method are particularly appropriate for the study of single-patients datasets, whose measurement quality can be affected to a different degree and at different site locations, making it difficult to statistically assess group-level effects. Moreover, in the context of intracranial data, the averaging of iEEG epochs rarely generates classical ERP components, thus preventing a straightforward use of the knowledge derived from scalp EEG studies. While it provides a stringent test for the classification of single-trial data together with an identification of anatomical sites for condition-dependent changes, this method also takes advantage of several single-trial features simultaneously, including signal amplitude and spatio-temporal pattern of activity (see *Hidden Markov Model of single-trial iEEG* section for details).

In the following, we first describe in details the HHM model estimation of single-trial iEEG and the accuracy estimation. We then systematically investigate the relation between the classification performance and the electrodes locations, as well as its relation with the parameters of the HMM models.

### Hidden Markov Model of single-trial iEEG

To model the neural response in the S-sequence, we used a selection of contacts for each of the two patients, choosing the most distant contacts on each electrode array. This data selection procedure was motivated by the need to reduce the computational time in training the model and is justified by the observation that close contacts measure highly correlated brain activity. The number of selected contacts *N* was 16 for M.R. and 22 for C.S. (for Talairach coordinates of these contacts, see Table S1). To achieve faster RTs, motor learning should predominantly modulate the electrical activity preceding the key press. We therefore defined each individual trial (corresponding to iEEG epochs) as starting from the fixation cross and ending when the key press was recorded. Because we aimed at characterizing neural responses when the patients performed the S-sequence, irrespective of the specific finger used, we pooled all the trials from the S-sequence in separate datasets, one for each of the two days. This approach of pooling together all the events corresponding to different fingers was also essential for allowing the use of the same model when classifying single trials of the C-sequence. Obviously, the model was not informed by any feature of the sequence order.

For each patient, we therefore considered two datasets one for day 1 and one for day 2 including a set of single trials (epochs) from the S-sequence. Each of these epochs comprises a set of voltage measurements M_tf_ at each time-frame *tf*, with M_tf_ = [v_1_(tf), v_2_(tf),…,v_N_(tf)] (Figure 2b,c). The aim of the HMM algorithm is to model the time series of vectors M_tf_ within each epoch {M_1_, M_2_,…, M_L_}, where L is the length of one epoch. Previous applications of HMM on electrophysiological data aimed at characterizing the sequential pattern of specific features extracted from the signal [27]–[29]. In our study we applied the HMM on the raw signal directly in order to characterize the sequence of voltage configuration across multiple sites of recordings that can subtend sequence learning.

In an HMM, one considers a probabilistic model of two sets of random variables {H_1_,…, H_Q_ } and {M_1_, M_2_,…, M_L_} [30]. The variables {H_1_,…, H_Q_ }, called hidden states, represent a stochastic process whose evolution over time cannot be observed directly. The property of this process can only be inferred through the realizations of the variables {M_1_, M_2_,…, M_L_}, that is to say, in our case, the EEG signal recorded from several electrodes. The HMM is characterized by the initial state probability vector π of elements π_i_ = P(H_1_ = i); the state transition matrix A, with a_ij_ = P(H_tf_ = i|H_tf–1_ = j) and the set of emission probability density function B = {b_i_(M_tf_) = p(M_tf_|H_tf_ = i)}, which is, in our case, modeled by a Gaussian mixture (Gaussian mixture model; GMM). The choice of a GMM for this probability density function is standard when HMM is applied to a continuous variable [30].

The first step of the algorithm is a GMM estimation of the set of voltage measurement for a given condition. Thus, the N-dimensional vectors {M_tf_}, representing the instantaneous voltage measurement across the entire set of electrodes (Figure 2b), were extracted and pooled together regardless of the timing at which they were observed and of the trial to which they belonged (Figure 2c). We initialized the GMM estimation itself by a K-means clustering method [31], which provided a first guess of the mean of each cluster, the prior probability for each of the clusters, and associated covariance. The latter were calculated considering, for each of the Q Gaussians, the set of vectors {M_tf_} closest (on the basis of Euclidean distance) to each mean. We estimated the GMM parameters by an expectation-maximization algorithm for mixture of Gaussians [32]. We used the resulting GMM parameters to initialize an HMM model with Q hidden states. In this second step we aimed at characterizing the temporal structure of the response as a series of states. The HMM model was estimated using Baum-Welch algorithm [33], an expectation-maximization procedure which estimates the HMM parameters by maximizing the probability of observation sequence given the model. Both steps in the model estimation were computed using a toolbox for Matlab written by Kevin Murphy (http://www.cs.ubc.ca/~murphyk/Software/HMM/hmm.html).

In order to select the number of clusters Q providing the best classification between the trials on day 1 and day 2, we considered a range of possible values of Q between 3 and 8. We therefore obtained six HMM (one model for each of the Q values considered) for each of the two datasets and each of the patients.

### Accuracy estimation

To select which HMM provided the best discrimination power between day 1 and day 2, we tested the models ten times. Each time, the S-sequence data was randomly divided into two parts: one training set, which was nine tenths of the data, and one testing set, which was the remaining one tenth. For each test, we computed a Receiver Operating Characteristic (ROC), whose underlying area provides an unbiased measure of the classification accuracy [34]. After selecting the parameters corresponding to the largest area lying beneath the ROC curve, we validated the models on a set of single trials belonging to the S-sequence that had not been previously used for the parameter selection (validation dataset). Moreover, we used the same models to compute the ROC curve as resulted from classifying single trials of the C-sequence as belonging to day 1 or day 2. A significantly lower performance in classifying trials belonging to the C-sequence would confirm that the classification performance between day 1 and day 2 for the S-sequence was attributable to sequence-specific effects related to learning and neural plasticity, and not to non-specific task adaptation effects (e.g. more efficient visuomotor mapping with practice) nor to general differences in measurement conditions (e.g. different levels of fatigue or motivation of the patient). For testing the model on the C-sequence, we considered ten splits of the whole set of single trials and ran a test for each split. It is worth noting that classifying isingle trials belonging to the C-sequence based on models estimated on the S-sequence is a sensible test because these models do not contain any information about the grammar of the sequence itself.

As an additional estimation of the quality of the trained model, we assessed the accuracy obtained for new test data by measuring the ratio of trials correctly classified and the total number of trials when keeping the same priors as those obtained during the training. Finally, to exclude that the discrimination power reached when classifying the S-sequence could trivially be due to shorter trial duration on day 2 compared to day 1, we performed a control test by classifying the two datasets based on the reaction times (RTs) only (i.e. based on the single-trial duration).

We compared the ROC curve areas based on reaction times and those based on the HMM models using the ten estimations in the cross validation procedure (t-tests; p<0.01). To further assess any possible relation between the estimated model accuracy and the RTs, we computed the Pearson correlation between the difference of the logarithm of the likelihoods of each of the two models (hereafter called discrimination function) and the RTs on a trial-by-trial basis in the test dataset.

### Estimating contacts with higher classification power

We aimed at testing whether the classification performance was equally driven by all the recording sites across the available electrode arrays or whether some sites might contribute more to the classification. We therefore dropped in turn each of the available electrode arrays, one at a time, and in each of the two subjects separately, and we recomputed the HMM models and the corresponding classification accuracy. At this stage, we considered each couple of electrodes together because they can reflect correlated activity along the strip. For each of the electrode selections, we estimated the parameters providing the highest classification accuracy for the S-sequence as we did above. We then compared the resulting classification performance with that obtained previously when keeping all the contacts. The same analysis was carried out with the data from the C-sequence based on the models estimated in the structure sequence.

A further analysis was carried out focusing on those couple of electrodes that produced a significant drop of the classification accuracy. In the following we refer to the string of contacts including this couple of electrodes as *target string*. In this further test, we trained two set of new models for day 1 and day 2 taking into account the same number of initially chosen selected contacts (i.e. 16 for M.R. and 22 for C.S.) but replacing one contact within the target string with another contact in the same string. The goal was to test how robust is the drop in classification accuracy against the different choice of the electrodes along the strip and the critical contribution of each single contact while keeping the same number of total electrodes.

### Relation between classification accuracy and parameters of the HMM models

One important step of multivariate decoding in neuroimaging studies is the identification of the features that the classifier exploits for achieving a good classification performance. We therefore investigated which parameter(s) from the HMM models was most informative in classifying single-trials from day 1 and day 2 and what signal properties did the relevant parameter(s) reflect. For each patient, we considered the set of HMM models (one model for each training dataset) estimated during day 1 and day 2. We considered here only the selected values of hidden variables providing the best accuracy (i.e. Q1 = 3, Q2 = 4 for patient M.R. and Q1 = 6, Q2 = 7 for patient C.S.). We investigated which parameters (i.e. priors, means, covariances, and transition probabilities), for each model was crucial for achieving a good classification performance. We ran an exhaustive analysis by considering all possible combinations for each set of parameters. For example for testing whether the means estimated from day 1 or day 2 were carrying critical information for classification performance, we swapped the values of the means of the first model with those of the second model considering all the possible permutation between the first and the second set. For each swap we assessed the accuracy and we evaluated whether there was a significant drop. The same analysis and the same tests for accuracy assessment (as described in the ‘Accuracy estimation’ section) were repeated for each set of parameters (i.e. priors, means, covariances, and transition probabilities).

Finally we aimed at finding a neurophysiological interpretability of the relevant parameters revealed by this procedure.

## Results

### Behavior

For each patient, we first averaged the reaction times (RT) over the 8 key presses of each sequence in each block (i.e. 20 sequences in each block). Note that only hits were included in the analysis and that the patients made very few errors (incorrect key pressed or misses) in each block (% of all key presses ±SD; Patient M.R.: 2±2; Patient C.S: 1±2). The data were then subjected to an ANOVA with Day (day 1, day 2) and Blocks (3 blocks for the S-sequence, 1 block for the C-sequence) as factors. Additional t-tests were performed whenever interactions were significant. Both patients showed a main effect of Day (patient M.R.: F(1,152) = 4.89, p<0.05; patient C.S.: F(1,152) = 15.72, p<0.001) due to the RTs being faster on the second than the first day (mean ±SD; M.R. Day 1: 597 ms±66 ms, Day 2: 575 ms±66 ms, T(158) = 2.11, p<0.05; C.S. Day 1: 401 ms±38 ms, Day 2: 379 ms±42 ms, T(158) = 3.38, p<0.001). Critically, the analysis revealed a Day by Block interaction (M.R.: F(3,152) = 3.62, p<0.05; C.S.: F(3,152) = 2.72, p<0.05) due to greater performance improvement for the S-sequence blocks than the C-sequence blocks on day 2 (RT difference between day 1 versus day 2 for M.R. S-sequence: 24 ms, T(118) = 2.21, p<0.05, C-sequence: 15 ms, T(38) = 0.72, p = 0.49; for C.S. S-sequence: 29 ms, T(118) = 5.16, p<0.001, C-sequence: −1 ms, T(38) = −0.07, p = 0.95). Consistent with previous studies [35], [36], none of the patients exhibited any explicit knowledge of the S-sequence or of fragmentary rule knowledge (i.e. three successive positions in the sequence). They reported that they did not notice any regularity in the succession of the yellow rectangles or key presses.

### Results from the multivariate decoding approach to single-trial iEEG

#### Classification accuracy results

**Figure 3:**
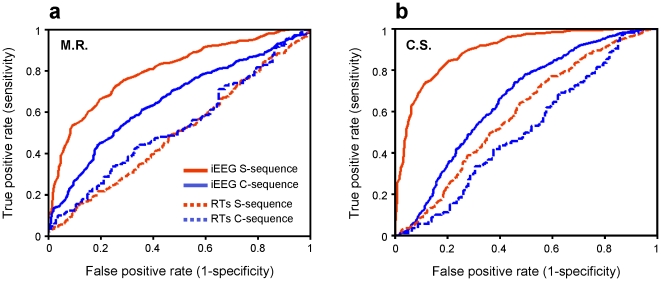
Classification results. ROC curves for M.R. (a) and C.S. (b) representing the classification results when taking into account all contacts, comparing the iEEG data from S-sequence (plain red line) and those from the C-sequence (plain blue line). The same model was used to classify reaction times from the S-sequence (dotted red line) and from the C-sequence (dotted blue line). Accuracy for classifying single-trials belonging to S-sequence was always significantly higher than that for the C-sequence and for the reaction times, suggesting that the classification algorithm captured information in the signal that was specific for the trained visuomotor sequence.

For patient M.R., the best classification performance was obtained for Q = 3 on day 1 and Q = 4 on day 2. The ROC area was 0.80±0.07 (mean ±SD; Figure 3a). For patient C.S., the selected parameters were Q = 6 and Q = 7 for day 1 and day 2, respectively. The average ROC curve area was 0.90±0.06 (Figure 3b). Importantly, we obtained the same results (indistinguishable ROC curves) when classifying single trials of the validation dataset which was not used for parameters selection. The accuracy obtained when keeping the same priors as those obtained in the training, measured as the ratio of the number of trials correctly classified to the total number of trials, was 0.70±0.06 and 0.81±0.05 respectively. In both patients, the accuracy for classifying the S-sequence was significantly higher than that for classifying the control sequence (p<0.01), because the average ROC curve area for the C-sequence was 0.66±0.04 and 0.66±0.04 for day 1 and day 2, respectively. The corresponding accuracy when keeping the same bias as in the training was 0.60±0.06 and 0.63±0.04. When classifying the trials in the S-sequence based on their reaction times only, we obtained a classification accuracy considerably lower than that for the S- and C-sequences in both patients (Figure 3). Note that, because both S- and C-sequences were recorded during the same sessions, we can exclude that the classification relied on other factors than sequence learning (such as simple visuomotor mapping, motivation, fatigue, etc.), which would have then resulted in a high accuracy for both sequences, instead of a high accuracy selectively for the S-sequence as found here.

In addition, the correlation between the discrimination function and the RTs evaluated at single-trial level and on each of the ten test datasets was also negligible and never exceeded 0.37 (n.s.).

### Contribution of the recording sites to the classification

**Figure 4:**
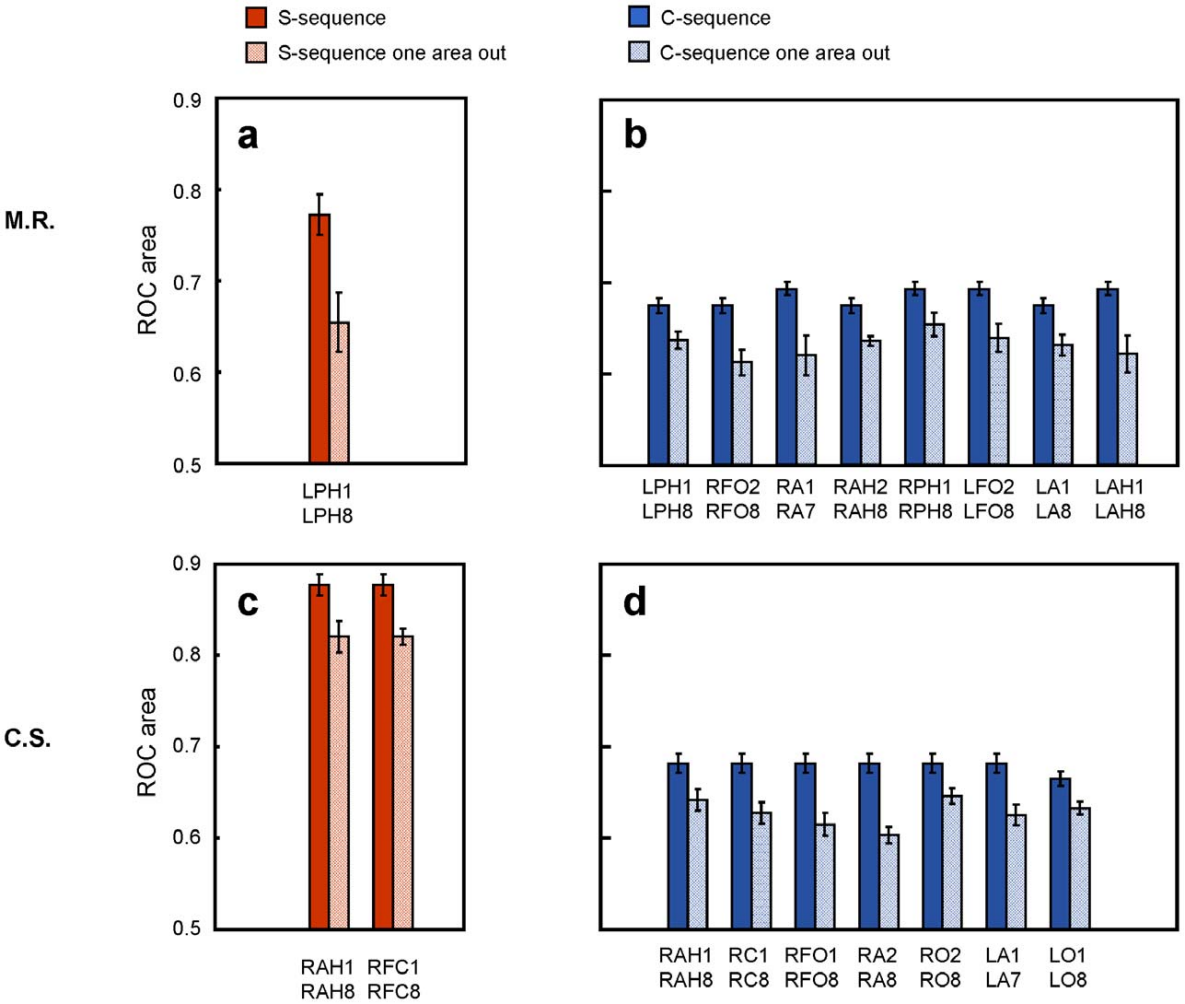
Localization results. ROC areas for M.R. (a) and C.S. (c), comparing classification performance when taking into account all implanted areas versus after dropping in turn one area. Red bars refer to the S-sequence, blue bars to the C-sequence (b and d). Significant differences between keeping all the contacts and dropping in turn couples of electrodes were observed for the S-sequence only for the string including the hippocampus and right frontal caudate contact for C.S. By contrast for the C-sequence, a significant drop in the classification accuracy was evident for many areas. Importantly single-trial classification remains always lower in the C-sequence than in the S-sequence. Abbreviations: LA = left amygdala; RA = right amygdala; LAH = left anterior hippocampus; LPH = left posterior hippocampus; RAH = right anterior hippocampus; RPH = right posterior hippocampus; LFO = left fronto-orbital area; RFO = right fronto-orbital area; LO = left occipital; RO = right occipital; RFC = right frontal-caudate. Standard errors are shown.

**Figure 5:**
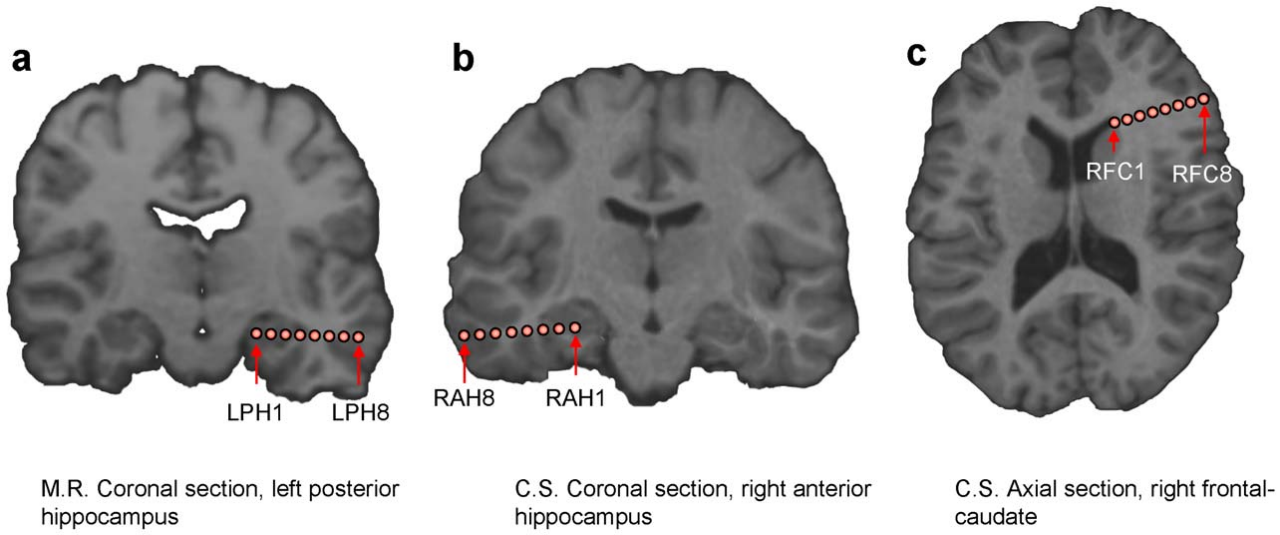
Visualization of the areas most contributing to the classification. (a) Left posterior hippocampus for patient M.R. (b) Right anterior hippocampus and (c) right frontal-caudate region for patient C.S. Electrodes localized by the CT scan were coregistered to the MRI T1-weighted brain volumes.

The accuracy for classifying the S-sequence was significantly (p<0.01) affected when we did not consider one electrode array in patient M.R. This array corresponded to left posterior hippocampus (Figure 4a and Figure 5a). We also found a significant drop in the accuracy for patient C.S. when we excluded the electrodes corresponding to either the right anterior hippocampus or the one corresponding to the right frontal-caudate array (Figure 4b and Figure 5b,c). We did not obtain a significant drop in the classification accuracy when excluding any of the other arrays. We report the results using model parameters that provided the highest classification performance for the S-sequence. Importantly, for none of the parameter values we obtained higher classification accuracy when excluding any of these areas. This last result provides a quality check on the contribution from each electrode included in the present analysis, because dropping an electrode with noisy signal might improve the classification accuracy.

When applying the same procedure on the data from the C-sequence, we found that, in both patients, many areas were associated with a significant drop in the accuracy when they were not included in the model estimation (Figure 4a,b) but, critically, the accuracy for the C-sequence always remained significantly lower than that of the S-sequence. Again, as for the S-sequence, removing one electrode never resulted in any increase in the discrimination power and this was true for any of the parameter sets chosen.

We further analyzed how specific was the contribution of the hippocampus for classifying single trials belonging to the S-sequence. We estimated two new sets of models for day 1 and day 2 by considering all the initially chosen contacts (i.e. 16 for M.R. and 22 for C.S.) but replacing the one in the hippocampus (LPH1 and RAH1 for M.R. and C.S. respectively) with one external to the hippocampus within the same target string. For patient M.R., the ROC area dropped to 0.78±0.07 which was significantly lower than the area obtained using all the initially chosen contacts (p<0.05). For patient C.S., the ROC curve area dropped to 0.88±0.04, significantly lower than when including the hippocampus (p<0.05).

We could conclude that for both patients within the string that included the hippocampus, the contact placed within the hippocampus was contributing more to the classification performance than other contacts on the same string. Note also that these effects were found for the hippocampal contacts in the hemisphere contralateral to the executing hand in both patients.

### Parameters of the HMM model underlying classification accuracy

We found that the values of the covariances were the most informative for discriminating day 1 and day 2 and that none of the other parameters did significantly impact the classification accuracy.

**Figure 6:**
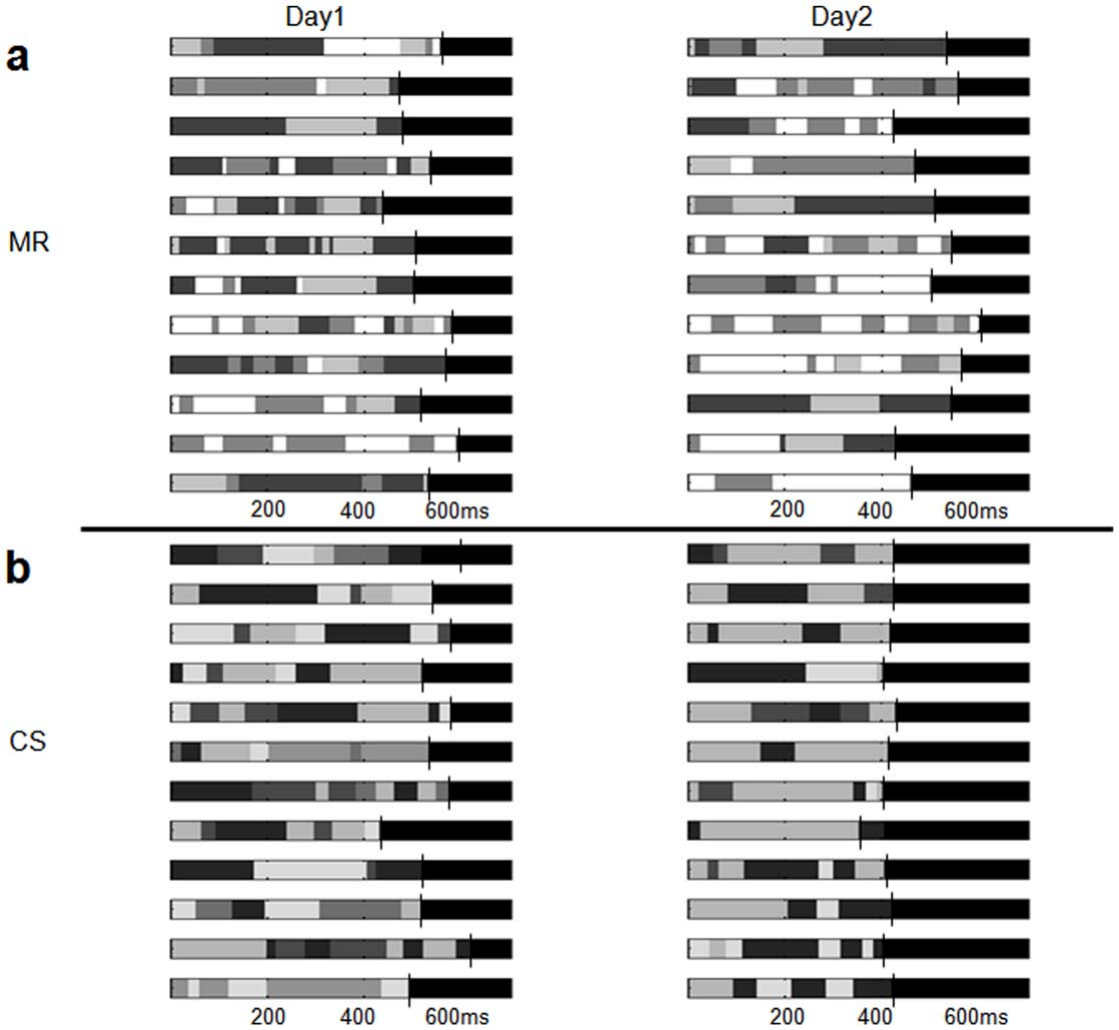
Segmentation of single-trials belonging to the structured sequence into voltage configurations (‘momentary states’). Each of these states is labeled in a gray scale gradation. The trials length (right-padded in black) is marked by a vertical line. For each subject (panel a and panel b), and each day (from left to right, day 1 and day 2 respectively), we show a series of exemplar single trials and the segmentation provided by the HMM algorithm. It should be noted that during day2 the average duration of these states is comparably longer that that during day 1 in both subjects. This observation was confirmed statistically in both subjects. The difference in duration length between day 1 and day 2 was highly significant and only during the structured sequence. This evidence provides new insight about spatio-temporal properties of neural activity underlying sequence-learning.

The next step was to gain a better insight on the neurophysiological interpretability of the HMM models' parameters estimated during day 1 and day 2. As mentioned above (section ‘Hidden Markov Model of single-trial iEEG’), the HMM model can be used to characterize the sequence of voltage configuration across multiple sites of recordings. Specifically, we used this model to segment single trials into series of momentary states, i.e. stable voltage configurations (Figure 6). We therefore investigated how differences in the covariance matrices (which were found above to play a significant role in the classification) may be reflected in the spatio-temporal pattern of these momentary states. We applied the two models providing the best classification results to each trial belonging to day 1 or day 2. We found that the average duration of these states was longer during day 2 and in comparison to day 1 only for those trials belonging to the S-sequence in both patients. It is worth noting that we always compared mean duration of the momentary states for each single trial obtained by applying the same model, and therefore the difference in mean duration was not due to different values of Q between the two sessions. The average duration of these momentary states for subject M.R. significantly differed between day1 and day2 (97 ms and 111 ms respectively; p<0.05; Figure 6). The average duration for subject C.S. was 63 ms and 71 ms during day 1 and day 2 (significantly different, p<0.05; Figure 6). Importantly, this difference in the average duration was no longer significant when considering single trials belonging to the random sequence in both subjects (during day 1 and day 2 average duration was 75 ms and 75 ms for M.R.; 60 ms and 64 ms for C.S.). We could conclude that the pattern of momentary states during the motor task and their typical temporal duration were modulated by the learning stage of the subject. In addition, when omitting the hippocampus contribution, for patient M.R. the difference in the average duration for the S-sequence was not anymore significant (61 ms and 64 ms during day 1 and day 2 respectively; p>0.05), and for patient C.S., although this difference was still significant, it was significantly lower than when including all the contacts (58 ms and 62 ms during day 1 and day 2 respectively; p>0.05).

## Discussion

Intracranial EEG recordings in humans provide spatially-localized measurements of brain activity with a high temporal resolution. In this study, we proposed an HMM-based multivariate algorithm that takes advantage of the distributed neural signals measured by iEEG at multiple recording sites. Although this approach can in principle be used to characterize the neural correlates of any cognitive functions, in this study we show that it can be successfully applied to track a visuo-motor learning effect. This dataset included recordings from two subjects who performed the task before and after a night of sleep. Without the subjects being aware of it, the two sessions included both a structured sequence (S-sequence) and a pseudo-random control sequence (C-sequence). Behavioral results from both patients showed that motor performance improved selectively for the S-sequence after a delay that included one night of sleep. These results are similar to what has been shown with previous SRTT and motor learning studies in normal subjects [37], [38], [39], and provide a strong indication that patients underwent learning-related neural changes in response to the structured sequence.

Consistently with the behavioral results, we showed that a multivariate classification algorithm can accurately assign single-trial iEEG responses to either the initial training phase (day 1) or to the later consolidated phase (day 2), i.e. after sequence-specific improvement occurred. Importantly, this classification result relied on activity patterns selectively associated to the learning of the regularity of the 8-item sequence (S-sequence), as evidenced by the comparatively low classification accuracy obtained for single-trials from the C-sequence. In addition we found that the classification failed when applied to the single-trial reaction-times, thus indicating that the learning-related changes captured by the decoding algorithm cannot be trivially explained in terms of speeding of motor response. Finally, while the localization of these learning-related changes involved a distributed pattern of neural activity, we identified the hippocampus as providing a major contribution to the reliable classification of the trained sequence, independently in each patient, with an additional contribution from frontostriatal regions for the patient implanted with electrodes in these brain areas.

Taken together, these results demonstrate the feasibility of the application of a multivariate approach for the analysis of learning-related changes in intracranial recordings in humans. This is mainly supported by the accurate performance of the classifier in tracking sequence learning at the level of single trials and by the highly consistent pattern of results obtained in the two patients analyzed independently. More specifically, independently in both patients (i) we could classify single trials based on distributed activity and only in relation to the S-sequence; (ii) the hippocampus provided the highest contribution to the classification accuracy compared to all the other sites; (iii) the distinct learning stages were paralleled by a difference in the average temporal duration of momentary neural states (see next section).

### Learning-related changes the stability of momentary neural states

The present study provides evidence of learning-related changes of intracranial electrical activity corresponding to single events (individual keypresses) belonging to a visuomotor sequence. The analysis of the features used by the classifier showed that the discrimination between different stages of the learning process relies on differences of the spatio-temporal patterns of the raw signal in relation to the learning stage. Specifically we showed that responses to single visual cues can be represented as a sequence of ‘momentary states’, whose duration changed as a function of learning, i.e. when comparing day 1 to day 2.

Indeed, this change in duration was observed only for trials belonging to the S-sequence, and critically related to the presence of the hippocampus in both subjects. The existence of short periods of stability of voltage configurations (often referred to as functional microstates) is a well-established empirical observation at the level of the scalp electroencephalography [40]–[43]. Their spatio-temporal properties have been extensively used to analyze both event-related potentials [44], [45] as well as resting state EEG [46]. Our findings demonstrate that the statistical properties of distributed voltage configurations are also detectable in intracranial responses at the single-trial level. The significant change in average duration of these momentary states suggests that the temporal properties rather than the actual amplitude values of these states are critically related to the learning stage. Variation of microstates' temporal duration has been reported at the level of scalp EEG in relation to functional deficits in specific clinical populations [47]–[49]. Very recently, it has been shown that resting state in humans is characterized by a multi-fractal temporal organization of the miscrostates whose degree of complexity is related to the temporal duration of the microstates [50]. These studies together with our current findings suggest that the temporal scale of momentary states carries crucial information about cognitive states emphasizing the importance of investigating the dynamics of brain activity together with its spatial distribution.

### Distributed versus localized neural correlates underlying sequence learning

Our results show that the underlying neural correlates of sequence learning is distributed within a large network, involving activities from multiple locations. However, in both subjects we found a significant drop in the performance only when activity recorded from the hippocampus (and from a frontostriatal region for the patient implanted with electrodes in these brain areas) was not included in the classification. These results suggest a major contribution in sequence learning of these regions, among the multiple sites at which electrodes were implanted. A more general conclusion about the specific role of the hippocampus and fronto-striatal activities is that sequence learning may require a larger population of patients. At present, these results speak in favor of the feasibility of the proposed algorithm; first in relation to the high consistency of these results across the two patients analyzed independently; second because the role of the hippocampus in sequence learning is consistent with similar findings in associative processes, including the integration of temporally- or spatially-organized information [51]–[58]. Also consistent with the present results, it has been suggested that the PFC may participate in the extraction of regularities based on internal representations, so as to improve behavioral control [59], [60]. Finally, in support of the role of hippocampal-prefrontal interactions in memory processes, Peyrache and al. (2009) [61] recently demonstrated that following the acquisition of new rules, PFC activity patterns reflected the neural patterns that occurred during the training phase [62], and that this reactivation occurred predominantly when hippocampal and cortico-hippocampal interaction was enhanced [63]–[69]. While this could only be observed in one patient, the present finding of a contribution of both the hippocampus and PFC activity to the classification of learning stages would be consistent with these findings.

### Advantages and limitations of multivariate decoding approach to single-trial iEEG

The present study demonstrates that the proposed multivariate decoding approach to single-trial iEEG datasets from individual patients offers an important methodological alternative to group studies of averaged data. Indeed, grouping iEEG data from different patients can be extremely challenging because the configuration of electrode placement varies across individuals and pathological conditions (e.g. epilepsy, Parkinson disease), and because iEEG data quality is often contaminated by artifacts at different recording sites. The restricted availability of such iEEG recordings is an additional factor motivating the development of methods that fully exploit individual patient datasets. Following a classification scheme, we made use of non-overlapping splits of the available data to validate differences in the temporal and spatial configuration of the signal. Such classification analysis exploits stimulus-related information extracted from distributed activity patterns (i.e. ‘pattern information analysis’) and avoids biases that may arise when hypotheses and inferential analyses are not independent [70] (e.g. selection of few electrodes in ERP studies). Pattern information analysis has predominantly been developed for neuroimaging studies based on fMRI [3]–[6]. Classification analysis of electrophysiological data has been predominantly developed in the context of Brain Computer Interface [71]–[74] studies while less effort has been devoted to its application in neuroimaging studies [7]. As illustrated by the present study, one main advantage of this methodological framework is that it does not require any *a priori* selection of the relevant neural sites or time-window of activity measurements. We complemented our approach by a localization procedure and showed that we can identify the regions contributing most to the performance of the classifier. Our results demonstrate that this methodological strategy is particularly adapted to test the effect of learning-related changes capturing task-related effects at the single-trial level, that are not time-locked to the visual cue or motor response, and do not require a fixed trial length.

The present findings could benefit from the simultaneous recording of scalp EEG which could allow a useful comparison with existing literature on the same topic [24], [75]. This will be the focus of future studies including a larger set of patients.

### Conclusions

Intracranial EEG recordings in humans provide a unique opportunity to investigate spatially localized neural activity with a high temporal resolution, in particular for deep regions that cannot be easily accessible to surface EEG recordings. These data can thus provide an intermediate level of observation linking animal cell-recording data and human neuroimaging findings. However, much like cell-recordings in animals, iEEG studies have a sparse distribution of recordings sites. In this study, we propose a multivariate decoding strategy to optimize the use of such distributed neural signals by allowing the full analysis of datasets from individual patients at the single-trial level and by offering an unbiased test for the contribution of individual electrodes to the observed effects.

## Acknowledgments

We thank Peter Dayan (University College London, UK) for insightful theoretical and methodological comments. We also thank Laurent Spinelli for technical support and the patients for participating in the study.

## Supporting Information

Material S1 **Sleep parameters.** Description of the sleep parameters of the night between the two recordings for each of the two patients.
(DOC)

Table S1 **Talairach coordinates of the implanted electrodes.** List of the normalized Talaraich coordinates of the implanted electrodes for each of the two patients. We report here all those coordinates that were included in the multivariate decoding analysis.
(DOC)
