# Supplementary material for: Decoding Sequence Learning from Single-Trial Intracranial EEG in Humans
Source: PLoS One. 2011 Dec 9;6(12):e28630. doi: 10.1371/journal.pone.0028630 (PMC3235148; doi:10.1371/journal.pone.0028630)
Supplement: Material S1 — Sleep parameters. Description of the sleep parameters of the night between the two recordings for each of the two patients. (DOC) [file pone.0028630.s001.doc]

**Supplementary Material S1**

Polysomnographic (PSG) recordings were scored by two trained sleep scorers (30-sec epochs), and from an initial 85% agreement, a final 100% agreement was reached after discussion of each non-consensual epoch. Sleep was scored based on scalp EEG (at position F1), measurements of horizontal and vertical eye movements, and chin electromyography. PSG scoring of the intracranial recording and quantitative analysis of the sleep parameters during the experimental night (between the training and test sessions) showed normal distribution of sleep stages, organized in four cycles for both patients and normal total sleep time (TST). In both patients, the percentage of stage 1 sleep remained within normal limits (< 10% TST). There was an increase in slow wave sleep (SWS; 53.52% TST for C.S. and 33.70% TST for M.R.) compared to age-matched normal values (15-20% TST) . REM sleep percentages (15.77% TST for C.S. and 25.34% TST for M.R.) were close to normal values (). Sleep architecture was little disturbed by wake periods in both patients (<30 awakenings/hour), and sleep stage shifts were frequent (>100/ whole night), as might be usually observed in such special clinical environments. However, sleep efficacy was good in both patients (>80%), which is particularly valuable for the present study. Consistent with the objective sleep parameters, the patients judged the quality of this night of sleep as good (St Mary’s Hospital Sleep Questionnaire and verbal assessment).

**Reference**

1. Billiard M, Dauvilliers Y (2005) Les troubles du sommeil. Paris: Masson.
