# Supplementary material for: Decoding Sequence Learning from Single-Trial Intracranial EEG in Humans
Source: PLoS One. 2011 Dec 9;6(12):e28630. doi: 10.1371/journal.pone.0028630 (PMC3235148; doi:10.1371/journal.pone.0028630)
Supplement: Table S1 — Talairach coordinates of the implanted electrodes. List of the normalized Talaraich coordinates of the implanted electrodes for each of the two patients. We report here all those coordinates that were included in the multivariate decoding analysis. (DOC) [file pone.0028630.s002.doc]

**Supplementary Table S2.**

| **a) Patient M.R.** | | | | | | | | |
| --- | --- | --- | --- | --- | --- | --- | --- | --- |
| **Talairach coordinates** | | | | | | | | |
|  | Medial contacts | | | | Lateral contacts | | | |
| Region | Name | x | y | z | Name | x | y | z |
| R frontal orbital | RFO2 | 10 | 22 | -22 | RFO8 | 41 | 31 | -13 |
| R amygdala | RA1 | 19 | -10 | -17 | RA7 | 50 | -3 | -26 |
| R ant. Hippoc. | RAH2 | 32 | -22 | -22 | RAH8 | 63 | -17 | -14 |
| R post. Hippoc. | RPH1 | 24 | -27 | -12 | RPH8 | 63 | -28 | -15 |
| L frontal orbital | LFO2 | -10 | 29 | -20 | LFO8 | -41 | 36 | -9 |
| L amygdala | LA1 | -23 | 00 | -19 | LA8 | -64 | 0 | -18 |
| L ant. Hippoc. | LAH1 | -23 | -20 | -20 | LAH8 | -64 | -14 | -18 |
| L post. Hippoc. | LHP1 | -21 | -30 | -10 | LHP8 | -62 | -27 | -10 |
| **b) Patient C.S.** | | | | | | | | |
| **Talairach coordinates** | | | | | | | | |
|  | Most medial contacts | | | | Most lateral contacts | | | |
| Region | Name | x | y | z | Name | x | y | z |
| R frontal orbital | RFO1 | 8 | 18 | -22 | RFO8 | 43 | 21 | -7 |
| R frontal caudate | RFC1 | 12 | 20 | 15 | RFC8 | 50 | 25 | 6 |
| R amygdala | RA2 | 22 | -10 | -23 | RA8 | 53 | -4 | -29 |
| R ant. hippoc. | RAH1 | 22 | -19 | -24 | RAH8 | 58 | -13 | -30 |
| R post. hippoc. | RPH1 | 28 | -33 | -15 | RPH8 | 64 | -28 | -22 |
| R occipital | RO2 | 31 | -56 | -6 | RO8 | 64 | -56 | -3 |
| L frontal | LF1 | -40 | 28 | 17 | LF5 | -54 | 31 | 13 |
| L amygdala | LA1 | -25 | -10 | -19 | LA7 | -57 | -7 | -25 |
| L ant. hippoc. | LAH3 | -22 | -17 | -18 | LAH8 | -50 | -17 | -15 |
| L post. hippoc. | LPH1 | -27 | -32 | -12 | LPH8 | -65 | -28 | -13 |
| L occipital | LO2 | -37 | -57 | -3 | LO8 | -70 | -53 | -0 |
| Note: Anatomical MRI scans were first normalized to the MNI template; the normalization parameters were then applied to the CT scans, on which the electrode contacts are visible; MNI coordinates corresponding to the electrode contacts were finally converted into Talairach coordinates. These coordinates are provided to illustrate the distribution of the recording sites, but do not replace the careful anatomical identification that we performed in native space.  Abbreviations: ant., anterior; hippoc., hippocampus; L, left; post., posterior; R, right | | | | | | | | |
